# Case Report: Clinical effect of eight plate epiphyseal block on leg length discrepancy in children: a retrospective study of 6 typical cases

**DOI:** 10.3389/fped.2026.1907249

**Published:** 2026-09-18

**Authors:** Yan Liu, Ningjing Zeng, Yang Lv, Guangxi Chen, Hongliang Liu, Jinfeng Li, Jiangfa Huang

**Affiliations:** 1 Department of Orthopedics, The Second Affiliated Hospital of Guangzhou University of Chinese Medicine, Guangzhou, Guangdong, China; 2 The Second Clinical Medical College, Guangzhou University of Chinese Medicine, Guangzhou, Guangdong, China; 3 Department of Anesthesiology, The Second Affiliated Hospital of Guangzhou University of Chinese Medicine, Guangzhou, Guangdong, China

**Keywords:** children, complication management, eight plate, epiphyseal block technique, limb length discrepancy

## Abstract

**Objective:**

This study aimed to investigate the clinical effect of epiphyseal block in treating children with LLD.

**Methods:**

The clinical data of 6 children with LLD who underwent epiphyseal block treatment in our hospital from March 2017 to April 2023 were collected and analyzed, including the preoperative and postoperative changes in the length of femur and tibia measured by full-length standing x-rays of both lower limbs, and the improvement of the unequal length of limbs after epiphyseal block using the “8” steel plate was understood and summarized. Treatment outcomes were descriptively evaluated according to the reduction in LLD, residual discrepancy after treatment, and treatment-related complications. Final residual LLD was classified as satisfactory correction (<15 mm), partial correction (15–20 mm), or limited correction (>20 mm).

**Results:**

The six patients (all male, aged 4–12 years, mean 7.83 ± 2.64 years) were treated for 12–45 months (mean 33.67 ± 11.91 months). Mean LLD decreased from 29.67 ± 7.76 mm preoperatively to 15.67 ± 9.42 mm after implant removal. No major complications occurred. Implant-related issues were managed differentially: loosening by hardware exchange; valgus by removing the lateral plate (retaining the medial); varus by removing the medial plate (retaining the lateral); retained hardware was removed after subsequent axis correction.

**Conclusion:**

Eight-plate epiphysiodesis is not a one-stage cure for pediatric LLD; larger discrepancies require longer treatment with more variables. Nevertheless, its minimally invasive nature makes it worthwhile with rigorous preoperative evaluation, skilled surgery, and close follow-up. Crucially, complications like loosening and axis deviations demand distinct management strategies to optimize outcomes.

## Introduction

Leg length discrepancy (LLD) is a common orthopedic condition in children, defined as a measurable difference in the lengths of the lower limbs. Such discrepancies can lead to gait disturbances, joint pain, and functional impairment, adversely affecting normal development and quality of life (1, 2). Effective interventions are therefore essential to mitigate these impacts.

In recent years, epiphyseal block procedures have been employed to correct LLD by modulating physeal growth, thereby achieving favorable outcomes (3, 4). The underlying principle involves applying controlled pressure on the growth plate to restrain its development. Al Badi et al. (5) reported that anterior epiphyseal fixation of the distal femur effectively corrects fixed knee flexion deformity in neuromuscular patients with a low complication rate. The technique has evolved from early U-shaped nails to the current eight-plate system (6). Both complete epiphysiodesis (growth arrest) and hemiepiphysiodesis (guided growth) are utilized to manage LLD and other lower extremity deformities during skeletal growth (7, 8). The eight plate is preferred for its minimally invasive nature and technical simplicity (9, 10). Braga et al. (11) confirmed the efficacy of epiphyseal fixation in adolescent lower limb deformities, and Zajonz et al. (12) highlighted its gentle and effective use for genu valgum. Compared with alternative methods, epiphyseal block offers less surgical trauma, shorter operative time, faster recovery, and a lower complication profile, making it particularly suitable for pediatric patients with LLD of 2–5 cm (13, 14).

Recent studies have further expanded the understanding of growth modulation techniques and their clinical applications. Starobrat et al. (15) reported that temporary epiphysiodesis using eight-plate implants could effectively regulate limb growth during LLD treatment, while also emphasizing the importance of continuous radiographic monitoring to identif*y* axis deviations and optimize timing of implant removal. Furthermore, advances in growth plate biology and regenerative medicine have highlighted the complex mechanisms underlying physeal injury, repair, and future therapeutic strategies. Rasouli and Wenke (16) summarized emerging approaches targeting growth plate regeneration, including biomaterial-based scaffolds, cellular therapies, and molecular modulation, suggesting new possibilities for improving long-term outcomes after physeal disorders. These advances indicate that while temporary epiphysiodesis remains an established clinical strategy, individualized treatment planning and long-term evaluation of growth-related complications remain essential.

However, most previous investigations have addressed epiphyseal block techniques without focusing on clinical outcomes in smaller case series that include detailed follow-up data. The present study contributes by offering a retrospective analysis of six cases, with particular attention to patient selection criteria, surgical difficulties, postoperative recovery, and specific complications encountered. Through this case series, we aim to delineate the variations in clinical response and to derive insights that may help refine surgical techniques for the benefit of future patients.

When evaluating treatment strategies for LLD, it is important to consider associated syndromic features. Conditions such as skeletal dysplasias and congenital syndromes can coexist with limb length inequality, and recognising these associations is essential for diagnosing and managing systemic comorbidities (17). For example, achondroplasia and Marfan syndrome may produce substantial discrepancies that require tailored therapeutic approaches. Only by acknowledging these syndromic contexts can clinicians formulate a comprehensive assessment and management plan for affected children.

Despite the potential advantages of epiphyseal blockade, clinical studies on its efficacy remain sparse. Further research is therefore needed to assess its effectiveness, stability, and complication profile. The present case series systematically examines the clinical outcomes of epiphyseal block therapy in paediatric LLD, providing a detailed analysis of six individual cases to explore treatment responses, interpatient variability, and adverse events. By investigating these specific cases, this study seeks to inform clinical decision-making, support appropriate patient selection, and add meaningful data to the existing literature on LLD management.

## Data and methods

### General clinical information

The clinical data of children with LLD who underwent treatment with eight plate epiphyseal block at our hospital from March 2017 to April 2023 were retrospectively collected and evaluated. Because this was a retrospective case series, potential selection bias and information bias could not be completely excluded. All eligible patients meeting the inclusion criteria during the study period were included. Complete clinical and radiographic follow-up data were available for all six patients, and no missing data requiring imputation were present. During the study period, six patients met the inclusion criteria, and all were male. This sex distribution reflected the characteristics of the eligible cases treated at our institution rather than a predefined sex-based selection criterion. Preoperative and postoperative femoral and tibial lengths were measured using full-length standing radiographs of both lower limbs. These measurements were analysed to assess the improvement in limb length inequality following epiphyseal block with the eight plate and to evaluate any complications related to the procedure. Given the small sample size, this work is best characterised as a case series. Although the findings are not broadly generalisable, the detailed case-by-case analysis can offer valuable insights into individual treatment responses, the surgical challenges encountered, and the postoperative outcomes in this patient group.

### Inclusion and exclusion criteria

The inclusion criteria were as follows: (1) children aged 3–14 years; (2) a leg length discrepancy (LLD) greater than 20 mm, as demonstrated on full-length standing radiographs of both lower limbs; and (3) treatment with an eight-plate epiphyseal block.

The exclusion criteria comprised: (1) patients with any form of physeal damage that had resulted in premature epiphyseal closure; (2) patients who had undergone other surgical procedures for LLD; and (3) patients with poor compliance who were unable to attend regular postoperative reviews and follow-up assessments.

### Patient selection and potential bias

Selection bias was minimized because all patients who met the inclusion criteria during the study period were included. However, as a retrospective single-center case series with a small sample size, selection bias and residual confounding cannot be completely excluded. Since all six patients had complete clinical records and radiographic follow-up, no missing data were observed, and therefore no statistical imputation methods were required. Given the rarity of qualifying cases, it was not possible to recruit additional patients beyond those treated during the specified study period.

### Surgical approach

The surgical procedure was performed according to previously described temporary epiphysiodesis techniques using tension-band plates (eight plates) for growth modulation in pediatric patients with limb length discrepancy and angular deformities (6, 9, 16). The operative strategy was based on established principles of guided growth, including accurate identification of the physis, appropriate positioning of screws across the growth plate, and regular postoperative radiographic monitoring. Minor adjustments regarding implant selection, screw positioning, and fixation management were made according to the patient's age, growth potential, deformity characteristics, and surgeon experience.

### Preoperative evaluation and preparation

Routine preoperative evaluations were performed, including blood tests, liver and kidney function tests, electrolyte panels, screening for infectious diseases, coagulation studies, chest radiography, and weight-bearing full-length standing radiographs of both lower limbs. For children with overgrowth of the affected limb, the contralateral shorter side was elevated to level the pelvis. Additional assessments comprised electrocardiography, cardiac ultrasonography, and routine urine and stool analyses. Only after all results were confirmed to be within normal limits was surgery scheduled; any abnormal findings that could affect the procedure were addressed prior to operation.

The number of eight plates to be implanted was determined based on the anatomical site of the length discrepancy, whether in the femur or tibia. Patient-specific factors, including age, weight, and bone development, were also considered when selecting the appropriate screw and plate sizes, with the aim of minimising the risk of postoperative screw loosening or breakage. Screw depth was limited to no more than half the epiphyseal width. Plate length was chosen to ensure that both screws would cross the physis, while maintaining a safe distance from the growth plate to avoid iatrogenic physeal injury. In children with severe deformity or high body weight, two eight plates could be placed simultaneously on the same epiphysis.

Before surgery, the surgical team communicated thoroughly with the family, explaining the patient's condition, the surgical technique, the associated costs, and the potential intraoperative and postoperative risks. Written informed consent and other relevant documentation were obtained to ensure the family's active cooperation throughout the treatment process.

### Operative technique

(1) The patient was placed in the supine position, and general intravenous anaesthesia was administered. An inflatable tourniquet was applied to the upper third of the thigh on the affected side. After routine sterilisation and draping, the limb was elevated and exsanguinated, and the tourniquet was inflated.
(2) A 1.0 mm Kirschner wire was positioned adjacent to the physis. The physeal location was identified under C-arm fluoroscopy and marked on the skin. A 2- to 3-cm longitudinal incision was then made, centred over the physis. The skin and superficial fascia were incised layer by layer, while the periosteum was left intact.
(3) A 1.0 mm or 1.5 mm guide wire was inserted parallel to the physis, as close as possible to its centre. C-arm views were obtained to confirm that the wire was positioned in the mid-lateral plane, avoiding excessive anterior or posterior placement to prevent growth in an undesirable direction and consequent deformity in other planes. The physis was carefully inspected before wire insertion, and the number of puncture attempts was minimised to reduce the risk of physeal injury.
(4) The eight plate was contoured according to the curvature of the bone surface to achieve better apposition. The central hole of the plate was placed over the end of the initial Kirschner wire, and two 1.5 mm Kirschner wires were inserted through the two peripheral holes. These guide wires were oriented as parallel as possible to the first positioning wire. Their position and direction were confirmed on both anteroposterior and lateral C-arm fluoroscopic images.
(5) Hollow self-tapping screws of appropriate length and diameter were then selected. The screw length did not exceed half the width of the physis. Two hollow screws were inserted over the two guide wires using a cannulated drill, and each screw was subsequently tightened. Final C-arm views were obtained to confirm proper placement of the plate and screws, after which the incision was closed in layers.

### Postoperative follow-up

After surgery, all patients were followed up at the outpatient clinic every three months. At each visit, full-length weight-bearing radiographs of both lower limbs were obtained. These imaging studies were used to measure the degree of deformity correction and to document the relevant parameters. Once satisfactory correction had been achieved, the implants were removed. Preoperative and postoperative imaging data were then compared to assess the overall therapeutic effect.

Owing to the small sample size, formal statistical testing was not performed. Instead, a descriptive case-by-case analysis was conducted to provide a detailed clinical interpretation of the outcomes. Imaging evaluation was based on the change in leg length discrepancy (LLD) from before surgery to after implant removal. The final residual LLD was categorised descriptively as good (less than 15 mm), moderate (15–20 mm), or poor (greater than 20 mm).

### Evaluation

Postoperatively, patients were followed up at the outpatient clinic every three months. At each visit, full-length weight-bearing radiographs of both lower limbs were obtained to evaluate deformity correction and to record relevant imaging parameters. Once satisfactory correction had been achieved, the eight-plate implants were removed. Changes in the imaging parameters before and after removal were compared to assess the overall treatment effect.

Treatment outcomes were evaluated based on the absolute change in limb length discrepancy (LLD) before and after treatment, residual LLD after implant removal or completion of treatment, and treatment-related events. Because this study was a small retrospective case series, statistical comparisons were not performed. For descriptive purposes, final residual LLD values were categorized as satisfactory correction (<15 mm), partial correction (15–20 mm), or limited correction (>20 mm). These categories were used only for descriptive reporting and do not represent validated clinical outcome definitions. In addition, implant-related complications, need for revision surgery, deformity progression, and recurrence were recorded and considered when interpreting individual patient outcomes.

## Results

The six children included in this study were all male, with ages ranging from 4 to 12 years (mean age, 7.83 ± 2.64 years). The mean treatment duration was 33.67 ± 11.91 months (range, 12–45 months), and follow-up ranged from 12 to 45 months. Detailed demographic and clinical characteristics are summarized in Table 1.

**Table 1:** General characteristics and clinical data of patients.

Case	Sex	Age at surgery (years)	Etiology of LLD	Affected site	Preoperative LLD (mm)	Treatment duration (months)	Follow-up duration (months)	Final residual LLD after treatment (mm)
1	Male	4	Left femoral fracture after internal fixation	Left distal femur + proximal tibia	32	36	36	21
2	Male	12	Lower limb length inequality	Right distal femur + proximal tibia	32	36	36	18
3	Male	7	Left femoral fracture after flexible nail fixation	Left distal femur + proximal tibia	23	12	12	20
4	Male	7	Lower limb length inequality	Left distal femur + proximal tibia	29	42	42	14
5	Male	8	Left lower limb shortening	Right distal femur + proximal tibia	41.98	45	45	12
6	Male	9	Left femoral fracture after plate fixation	Left distal femur	20	30	30	0

LLD, limb length discrepancy. This table summarizes the demographic characteristics, etiologies, anatomical sites, baseline LLD, treatment duration, follow-up duration, and final residual LLD of the six included patients.

The mean preoperative LLD was 29.67 ± 7.76 mm. After completion of treatment and implant removal, the mean residual LLD decreased to 15.67 ± 9.42 mm, corresponding to a mean correction of 14.00 ± 5.62 mm.

Treatment responses varied among individual patients (Table 2). The correction of LLD ranged from 3 to 29.98 mm. Three patients achieved satisfactory correction with a final LLD <15 mm, whereas the remaining patients showed partial or limited correction. During follow-up, fixation-related complications occurred in several patients. For implant loosening or malposition, the hardware was exchanged for new implants. For progressive knee valgus or varus deformities, the medial or lateral plate was selectively removed (medial plate removal for varus, lateral plate removal for valgus) to allow spontaneous correction, with the remaining hardware removed after the deformity had resolved. No major complications such as infection or neurovascular injury were observed.

**Table 2:** Clinical outcomes and complications after eight plate epiphyseal block treatment.

Case	LLD before treatment (mm)	Final LLD after treatment (mm)	LLD correction (mm)	Fixation status	Outcome classification	Complications and additional interventions
1	32	21	11	Removed after correction	Limited correction	Progressive knee valgus occurred during treatment; the lateral tibial plate was removed while the medial plate was retained. After valgus improved, the remaining hardware was removed. Femoral implants were removed earlier due to complete correction of femoral length.
2	32	18	14	Removed after correction	Partial correction	Implant loosening and malposition occurred; the loosened screws and plate were exchanged for new implants. Subsequent knee valgus required removal of the lateral tibial plate; the medial plate was retained until valgus resolved, after which all remaining hardware was removed at the parents' request.
3	23	20	3	Removed early	Limited correction	Limited effect of the eight plate epiphyseal block; treatment was discontinued by the family and implants were removed at another hospital
4	29	14	15	Removed after satisfactory correction	Satisfactory correction	Implant loosening and positional changes required exchange of the loosened hardware for new implants. No axis deviation occurred; after satisfactory correction, all implants were removed.
5	41.98	12	29.98	Removed after correction	Satisfactory correction	Initial valgus deformity was managed by removal of the lateral femoral and tibial plates, with medial plates retained. Subsequent varus shift required removal of the medial plates. Hardware was exchanged on one occasion due to loosening.
6	20	0	20	Removed after complete correction	Satisfactory correction	No complications observed

LLD, limb length discrepancy. Outcome classifications were used only for descriptive reporting in this case series according to final residual LLD: satisfactory correction (<15 mm), partial correction (15–20 mm), and limited correction (>20 mm). These categories were not intended as validated clinical outcome scores. Fixation status indicates the final condition of the eight plate implants after completion or interruption of treatment.

### Case 1

This patient achieved a good overall outcome, although the clinical course was complicated by the successive development and subsequent correction of knee valgus. The preoperative leg length discrepancy (LLD) was 32 mm (Figure 1A). At 10 months postoperatively, the LLD had decreased to 25 mm, and mild valgus of the left knee was noted (Figure 1B). By 2 years after surgery, the LLD was 8 mm, and femoral lengths were equal; however, there was evidence of progressive knee valgus. Consequently, the femoral internal fixation was removed, and the tibial fixation was replaced both medially and laterally (Figure 1D). At 2 years and 4 months postoperatively, the LLD measured 13 mm, and the knee valgus had worsened substantially (Figure 1E), prompting removal of the tibial hardware (Figure 1F). At 2 years and 7 months, the LLD was 18 mm, and the left knee valgus had improved (Figure 1G). By 3 years after the initial surgery, the LLD was 21 mm, and the knee valgus had resolved completely; all remaining internal fixation was removed in the same month (Figure 1H).

**Figure 1:**
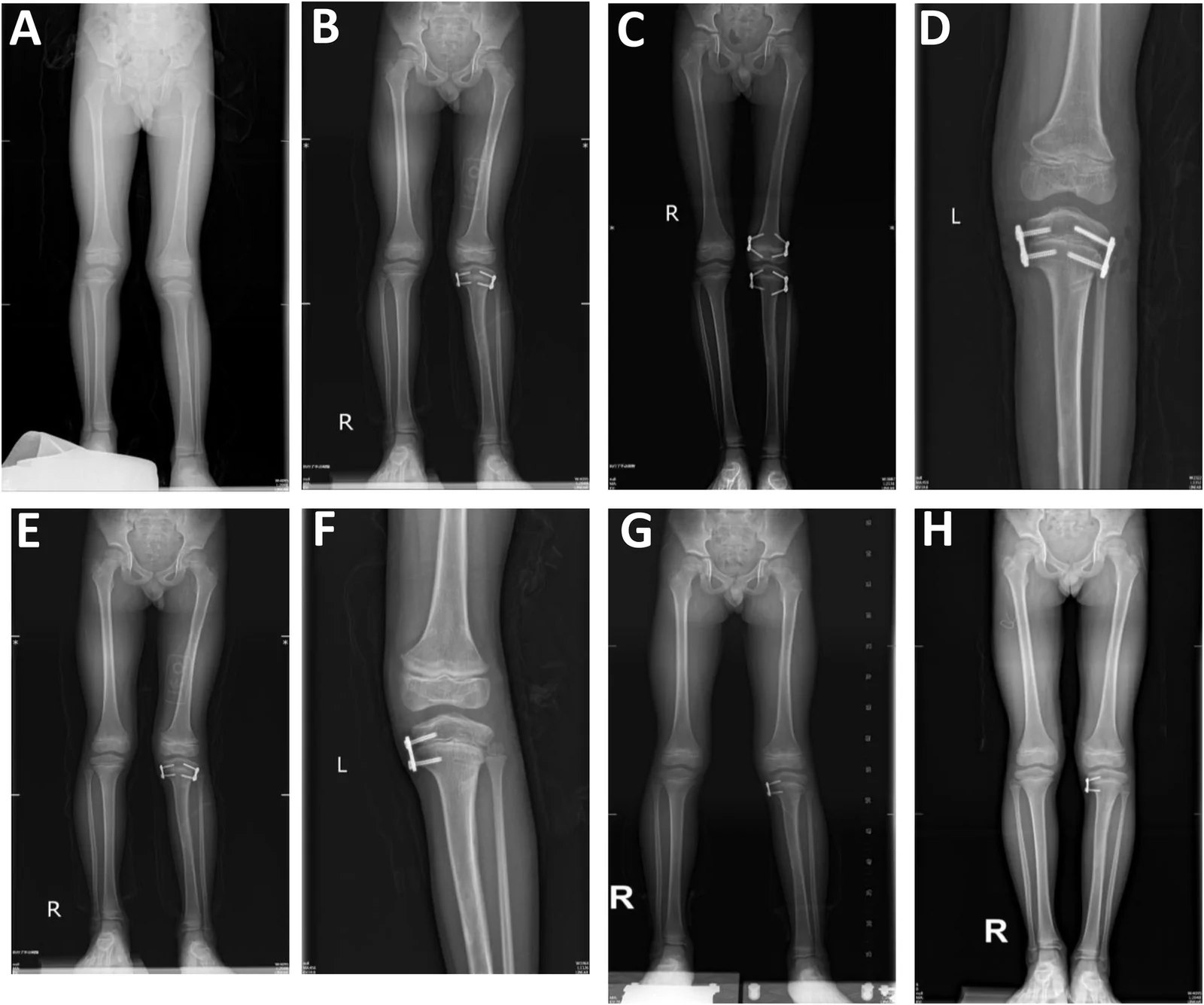
Treatment history of case 1. Sequential radiographs demonstrating the correction of limb length discrepancy (LLD) and the onset and resolution of knee valgus over the treatment period. **(A)** Preoperative radiograph showing an LLD of 32 mm. **(B)** Radiograph obtained 10 months postoperatively, showing a reduction in LLD to 25 mm and mild left knee valgus. **(C)** Radiograph obtained approximately 2 years after surgery, showing an LLD of 8 mm and equal femoral lengths, with progressive knee valgus. **(D)** Radiograph obtained after removal of the femoral internal fixation and revision of the tibial fixation. **(E)** Radiograph obtained 2 years and 4 months postoperatively, showing an LLD of 13 mm and substantial progression of knee valgus. **(F)** Radiograph obtained after removal of the tibial hardware because of progressive knee valgus. **(G)** Radiograph obtained 2 years and 7 months postoperatively, showing an LLD of 18 mm and improvement of knee valgus. **(H)** Radiograph obtained 3 years after the initial surgery, showing an LLD of 21 mm and complete resolution of knee valgus, with removal of the remaining internal fixation.

### Case 2

This patient achieved only partial correction and experienced worsening of knee valgus as well as implant loosening during the treatment course. The preoperative LLD was 32 mm (Figures 2A,B). At 6 months postoperatively, the LLD measured 27 mm (Figure 2C), and at 1 year, it had decreased to 20 mm (Figure 2D). At 1.5 years postoperatively, the LLD was 13 mm; however, implant loosening and malposition were noted, and all hardware was therefore revised and replaced during the same month (Figure 2E). At 2.5 years postoperatively, the LLD had reduced to 7 mm, but this was accompanied by aggravated knee valgus (Figure 2F), prompting removal of the medial proximal tibial plate. At the 3-year follow-up, the residual LLD was 18 mm, with mild persistent tibial valgus (Figure 2G, H). Complete hardware removal was subsequently performed at the parents' request.

**Figure 2:**
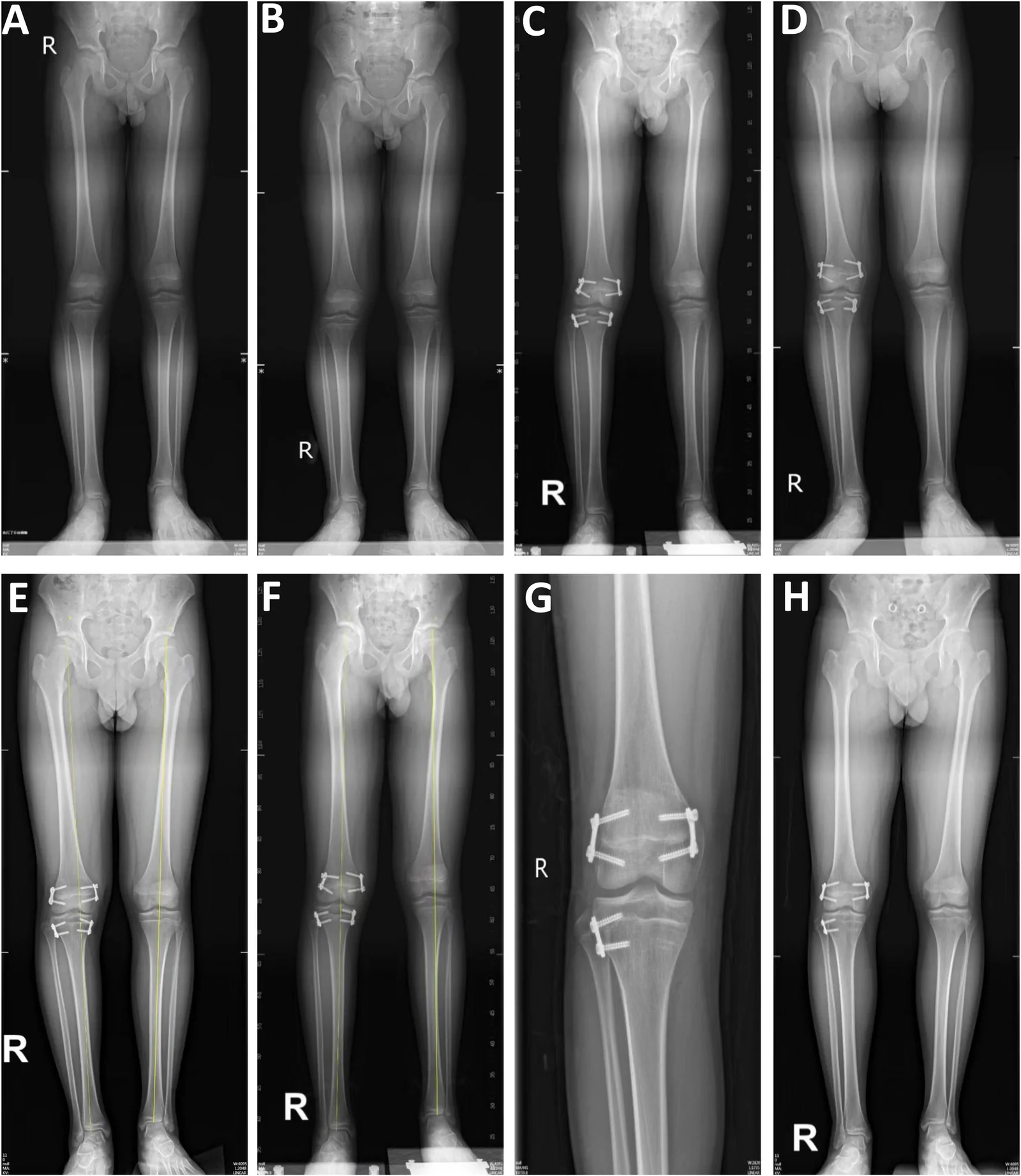
Treatment history of case 2. Sequential radiographs demonstrating changes in limb length discrepancy (LLD), knee alignment, and internal fixation during treatment. **(A, B)** Preoperative full-length lower-limb and knee radiographs showing a baseline LLD of 32 mm. **(C)** Radiograph obtained 6 months postoperatively, showing an LLD of 27 mm. **(D)** Radiograph obtained 1 year postoperatively, showing a reduction in LLD to 20 mm. **(E)** Radiograph obtained 1.5 years postoperatively, showing an LLD of 13 mm with implant loosening and malposition, followed by hardware revision and replacement. **(F)** Radiograph obtained 2.5 years postoperatively, showing an LLD of 7 mm with aggravated knee valgus, requiring selective adjustment of the tibial fixation. **(G, H)** Radiographs obtained at approximately 3 years postoperatively, showing a residual LLD of 18 mm with mild persistent tibial valgus and the subsequent treatment status before complete hardware removal.

### Case 3

No complications were observed in this case. However, treatment was discontinued prematurely because the parents were dissatisfied with the clinical response. Although the postoperative epiphyseal block appeared to be effective in reducing the preoperative LLD of 23 mm, the family considered the correction insufficient and subsequently had the internal fixation removed at another hospital.

### Case 4

Although this patient presented with an apparent varus deformity of the knee, the overall treatment outcome was satisfactory. The preoperative LLD was 29 mm (Figure 3A). One year after surgery, the internal fixation was replaced because of positional changes and evidence of implant loosening (Figure 3B). At two years postoperatively, the LLD had decreased to 20 mm (Figure 3C), and by three and a half years, it had further reduced to 14 mm (Figure 3D). The parents were satisfied with the clinical response; however, owing to financial constraints, they requested complete removal of the internal fixation, which was carried out as planned (Figure 3E).

**Figure 3:**
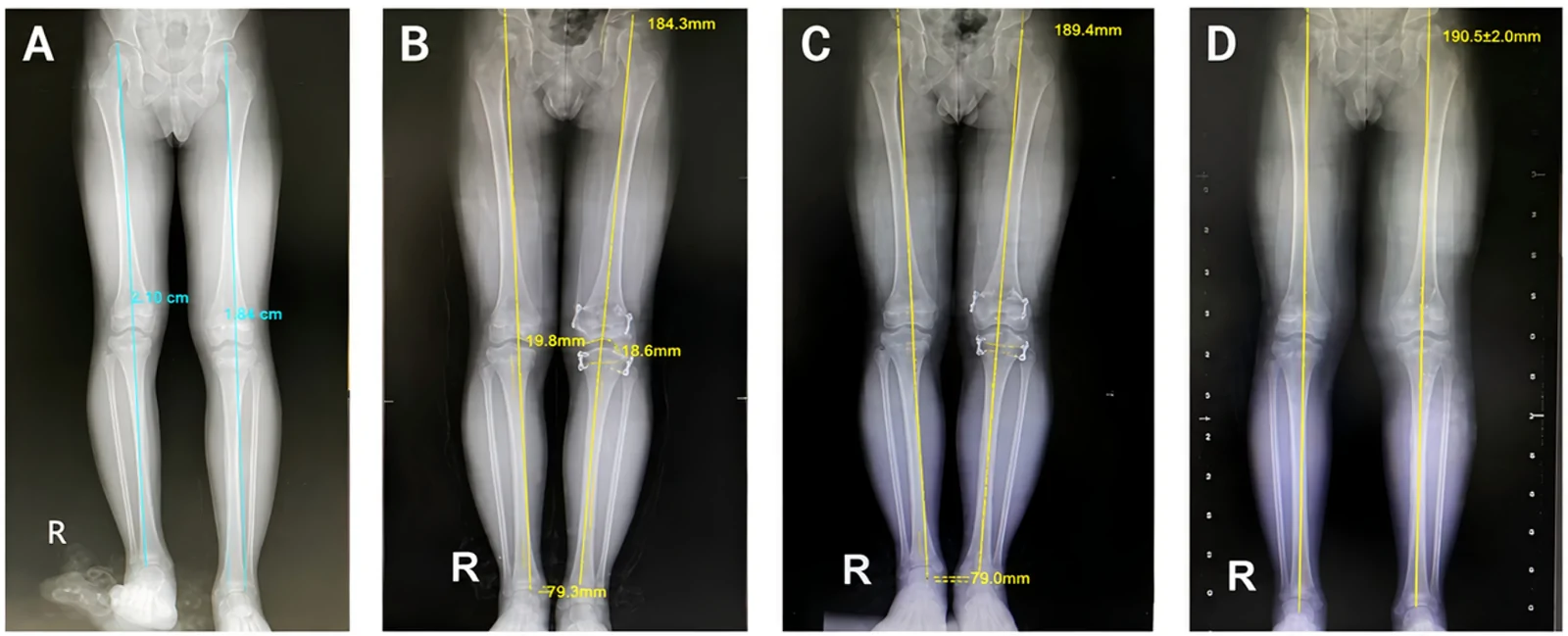
Treatment history of case 4. Serial radiographs demonstrating the treatment course, internal fixation replacement, and gradual reduction in limb length discrepancy (LLD). **(A)** Preoperative radiograph showing an LLD of 29 mm. **(B)** Radiograph obtained 1 year postoperatively, showing positional changes and implant loosening, which required replacement of the internal fixation. **(C)** Radiograph obtained 2 years postoperatively, showing a reduction in LLD to 20 mm. **(D)** Radiograph obtained 3.5 years postoperatively, showing a further reduction in LLD to 14 mm and satisfactory correction.

### Case 5

In this case, the epiphyseal block effect was pronounced, with the LLD decreasing from 41.98 mm preoperatively to 12 mm at final follow-up. However, the clinical course was characterised by an initial progression of valgus deformity followed by a subsequent varus shift. The preoperative LLD was 41.98 mm (Figure 4A). At one and a half years after surgery, the LLD measured 38 mm (Figure 4B), and the internal fixation was replaced because of diminished mechanical efficacy. At two and a half years postoperatively, the LLD had decreased to 18 mm, and a right knee valgus deformity began to emerge (Figure 4C). By three years after surgery, the LLD was 12 mm (Figure 4D), but the right knee valgus had worsened further. The lateral internal fixation was therefore removed (Figure 4E). At three years and nine months postoperatively, a right knee varus deformity developed (Figure 4F). The medial plate was subsequently exchanged for a lateral blocking plate. At the most recent follow-up, the LLD remained at 12 mm, and the patient continues to be monitored.

**Figure 4:**
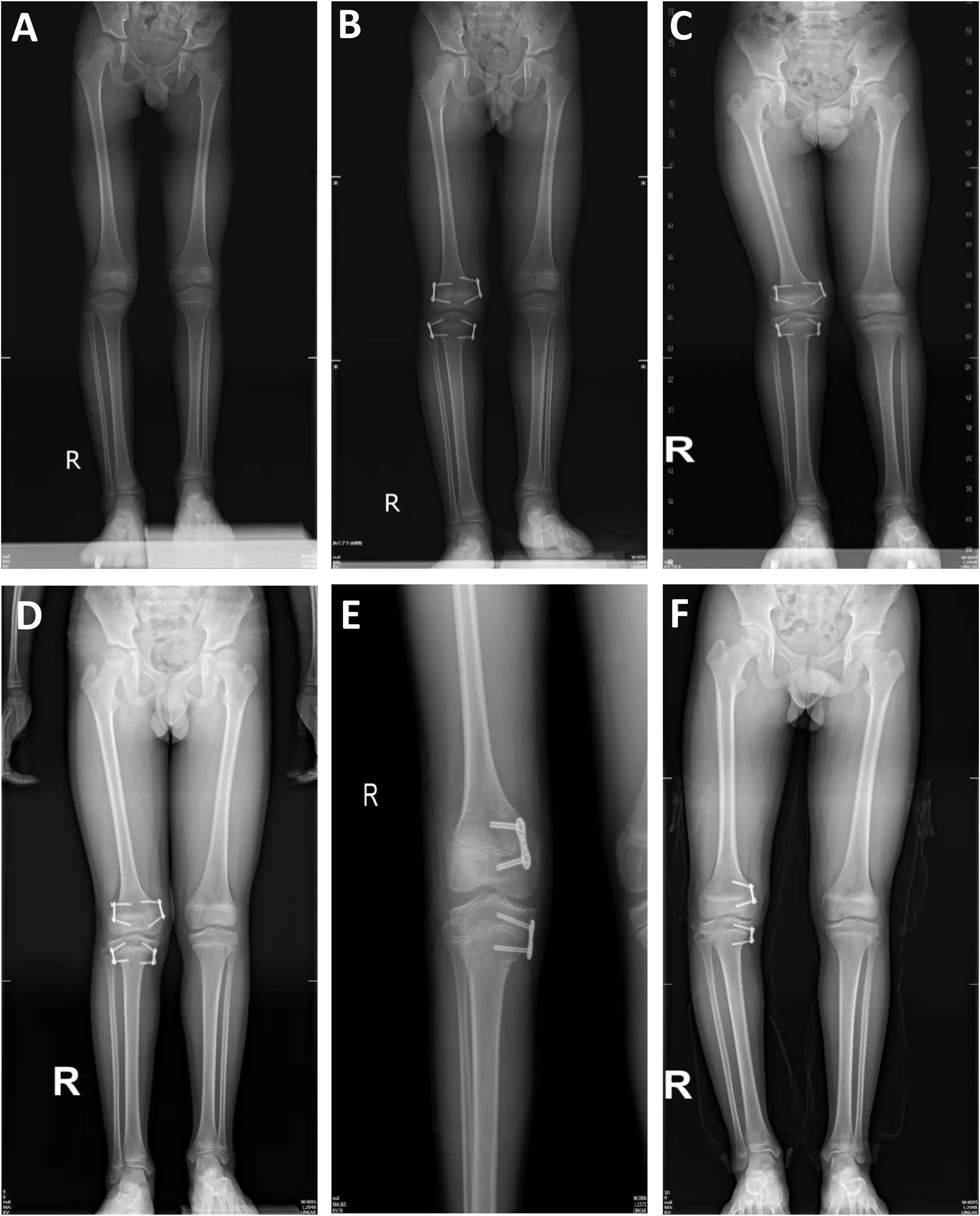
Treatment history of case 5. Sequential radiographs illustrating substantial reduction in limb length discrepancy (LLD), adjustment of internal fixation, and progression from knee valgus to knee varus during treatment. **(A)** Preoperative radiograph showing an LLD of 41.98 mm. **(B)** Radiograph obtained 1.5 years postoperatively, showing an LLD of 38 mm and replacement of the internal fixation because of diminished mechanical efficacy. **(C)** Radiograph obtained 2.5 years postoperatively, showing a reduction in LLD to 18 mm and the emergence of right knee valgus. **(D)** Radiograph obtained 3 years postoperatively, showing an LLD of 12 mm with progression of right knee valgus. **(E)** Radiograph obtained after removal of the lateral internal fixation to manage progressive valgus deformity. **(F)** Radiograph obtained 3 years and 9 months postoperatively, showing development of right knee varus deformity and subsequent adjustment of the internal fixation.

### Case 6

This patient was treated over a period of two and a half years. The LLD was fully corrected, decreasing from 20 mm preoperatively to 0 mm at the final follow-up. The preexisting varus deformity of the knee was also corrected simultaneously. No complications were observed throughout the treatment course (Figure 5).

**Figure 5:**
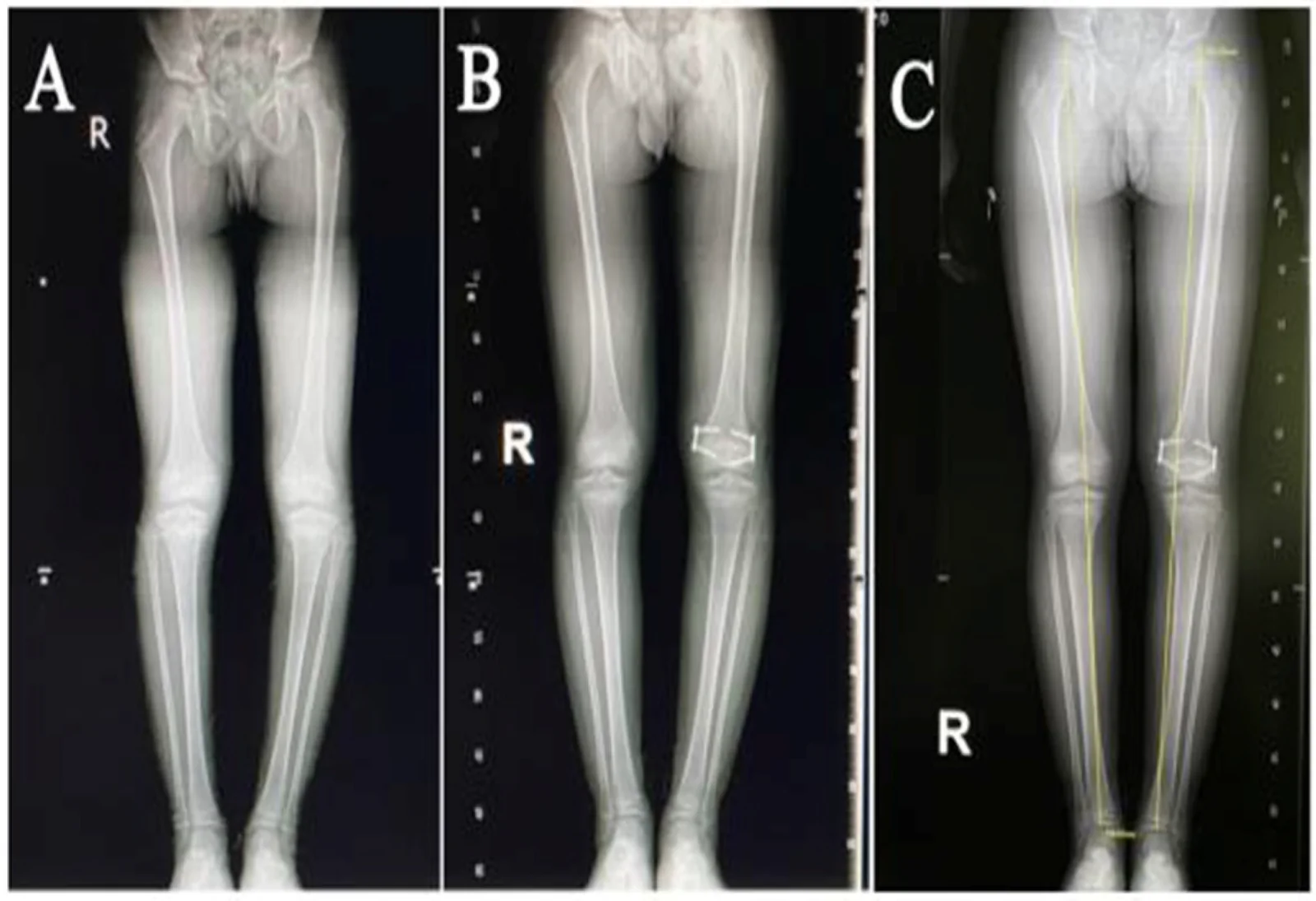
Treatment history of case 6. Sequential radiographs demonstrating complete correction of limb length discrepancy (LLD) and the associated genu varum deformity following treatment. (A) Preoperative radiograph showing an LLD of 20 mm and associated genu varum deformity. (B) Radiograph obtained during treatment showing temporary epiphysiodesis using eight-plate fixation. (C) Final follow-up radiograph showing complete correction of the LLD to 0 mm and correction of the associated genu varum deformity following treatment completion.

### Summary of findings

This study presents LLD changes as absolute values rather than according to any arbitrary grading system, with the aim of ensuring a more standardised and objective assessment. The reductions in LLD observed with epiphyseal block therapy were variable across cases, and several complications were encountered, including implant loosening, secondary axial deviations, and the need for hardware revision. Future studies should seek to validate a standardised classification framework, which would promote greater consistency in outcome reporting and enable more meaningful comparisons with findings from larger cohorts.

## Discussion

This retrospective case series evaluated the efficacy of temporary epiphysiodesis with the eight plate in six children with leg length discrepancy. The mean LLD decreased from 29.67 mm preoperatively to 15.67 mm after implant removal, with individual final LLD values ranging from 0 to 21 mm. Only three patients achieved a final LLD below 15 mm, yet no major complications such as neurovascular injury or deep infection were observed. These findings confirm that the procedure can produce meaningful reductions in LLD, although the outcomes were variable and not uniformly excellent. The technique therefore remains a viable option, consistent with earlier descriptions of epiphyseal block procedures (3, 4).

Our results are broadly consistent with previous reports. Demirel et al. observed a reduction from 39 mm to 22.4 mm in a similar cohort using the same device (18). Zajonz et al. also confirmed the effectiveness of eight-plates for correcting knee valgus, with most patients achieving satisfactory alignment (12). The slightly larger residual discrepancy in our series may be explained by heterogeneity in patient age, aetiology, and remaining growth potential. Post-traumatic shortening, which predominated in our cases, often follows a less predictable course than congenital or idiopathic LLD, and this may have contributed to the moderate correction rate observed.

Several alternative treatment options exist for managing LLD. Distraction osteogenesis can achieve substantial lengthening but is associated with prolonged treatment and higher complication rates (19). Guided growth using tension-band plates is less invasive, yet its success depends heavily on precise timing and appropriate patient selection (20). Percutaneous epiphysiodesis offers simplicity but may not adequately correct discrepancies exceeding 20 mm (21). Notably, a comparative study of percutaneous screws, plates, and staples found no significant differences in final limb length or correction rates, suggesting that device choice may be less critical than proper application (22). In this context, the eight plate remains an attractive option for moderate LLD because of its technical ease and low morbidity.

Complications observed in this series warrant careful attention. We encountered two distinct types of complications requiring different management strategies. Implant loosening or malposition was managed by exchanging the loosened screws and plate for new implants. Secondary knee valgus or varus deformities developing during treatment required a different approach: for valgus deformity, the lateral plate was removed while the medial plate was retained; for varus deformity, the medial plate was removed while the lateral plate was retained. After the axis deviation had resolved spontaneously, the remaining hardware was subsequently removed. These issues often necessitated secondary procedures, thereby interrupting the treatment course and increasing the surgical burden (23–25). Importantly, the management of axis deviations differs fundamentally from that of implant loosening, and the two should not be conflated as a single “exchange” procedure. The development of coronal plane deviations underscores the importance of precise screw placement and the avoidance of eccentric physeal tethering. Furthermore, recent evidence suggests that core stability may influence knee valgus during weight-bearing (25), and variations in fixation stiffness between the femur and tibia could affect the rate of correction (26). Therefore, a tailored approach that accounts for skeletal maturity, deformity pattern, and regular radiographic monitoring at three-month intervals is essential to enable timely detection and management of axis changes. Importantly, these implant-related complications require distinct management strategies rather than a uniform approach. Differentiating between loosening and axis deviations is critical: loosening warrants hardware exchange, whereas axis deviations (valgus or varus) are managed by selective unilateral plate removal, with the contralateral plate retained until spontaneous correction occurs and then removed subsequently. This distinction has direct implications for surgical decision-making and follow-up planning.

Several limitations should be acknowledged. This was a retrospective, single-center case series with a small sample size, which limits statistical power and generalizability. Although all included patients had complete clinical and radiographic follow-up without missing data, retrospective data collection may still introduce selection bias and unmeasured confounding. Therefore, the findings should be interpreted cautiously and require validation in larger prospective multicenter studies. All patients were male, not because of any selection bias, but due to the distribution of eligible cases during the study period; nevertheless, our findings may not fully apply to female children. The absence of a control group precludes direct comparison with other surgical methods. In addition, the follow-up duration was relatively short for some patients, and long-term outcomes after physeal closure remain unknown. Future multicentre prospective studies with larger and more balanced cohorts are needed to validate our observations and refine treatment algorithms.

Despite these limitations, our study provides valuable granular data on the real-world challenges associated with epiphyseal block therapy, including fixation failures, secondary deformities, and the need for repeated interventions. Such details are often overlooked in larger series but are critical for informed clinical decision-making. We conclude that temporary epiphysiodesis using the eight plate is not a single corrective procedure; rather, it initiates a dynamic process in which the initial LLD magnitude, treatment duration, and individual growth response collectively determine the final outcome. The procedure is minimally invasive and generally well tolerated, but its success depends on meticulous preoperative planning, skillful surgical execution, and rigorous postoperative surveillance. Our experience supports the cautious use of this technique for moderate LLD, while emphasizing the need for continued research into optimized surgical strategies and long-term management protocols.

## Conclusion

Temporary epiphysiodesis using the eight plate can effectively reduce leg length discrepancy in selected children, and its minimally invasive nature offers a clear advantage over more extensive procedures. However, this technique does not guarantee equalisation in a single operation. The magnitude of the initial discrepancy, the duration of treatment, and the individual growth response collectively determine the final outcome, rendering the process inherently variable. Complications such as implant loosening, secondar*y* axis deviations, and rebound deformities may occur and require timely recognition and appropriate management. It is important to distinguish between these conditions: loosening warrants implant exchange, whereas axis deviations (valgus/varus) are managed by selective removal of the unilateral plate (lateral removal for valgus, medial removal for varus), with the contralateral plate retained until correction is achieved and subsequently removed. Successful application therefore depends on meticulous preoperative assessment, precise surgical execution, and regular postoperative surveillance. Although the eight-plate epiphyseal block is a worthwhile option for moderate LLD, clinicians should remain aware of its limitations and the need for individualised treatment planning. Future studies with larger cohorts and longer follow-up are essential to further refine patient selection and optimise the timing of implant removal.

## Data Availability

The raw data supporting the conclusions of this article will be made available by the authors, without undue reservation.
